# Astrocyte-Specific Overexpression of Insulin-Like Growth Factor-1 Protects Hippocampal Neurons and Reduces Behavioral Deficits following Traumatic Brain Injury in Mice

**DOI:** 10.1371/journal.pone.0067204

**Published:** 2013-06-27

**Authors:** Sindhu K. Madathil, Shaun W. Carlson, Jennifer M. Brelsfoard, Ping Ye, A. Joseph D’Ercole, Kathryn E. Saatman

**Affiliations:** 1 Spinal Cord and Brain Injury Research Center, University of Kentucky, Lexington, Kentucky, United States of America; 2 Department of Pediatrics, University of North Carolina at Chapel Hill, Chapel Hill, North Carolina, United States of America; Max Planck Institute of Psychiatry, Germany

## Abstract

Traumatic brain injury (TBI) survivors often suffer from long-lasting cognitive impairment that stems from hippocampal injury. Systemic administration of insulin-like growth factor-1 (IGF-1), a polypeptide growth factor known to play vital roles in neuronal survival, has been shown to attenuate posttraumatic cognitive and motor dysfunction. However, its neuroprotective effects in TBI have not been examined. To this end, moderate or severe contusion brain injury was induced in mice with conditional (postnatal) overexpression of IGF-1 using the controlled cortical impact (CCI) injury model. CCI brain injury produces robust reactive astrocytosis in regions of neuronal damage such as the hippocampus. We exploited this regional astrocytosis by linking expression of hIGF-1 to the astrocyte-specific glial fibrillary acidic protein (GFAP) promoter, effectively targeting IGF-1 delivery to vulnerable neurons. Following brain injury, IGF-1Tg mice exhibited a progressive increase in hippocampal IGF-1 levels which was coupled with enhanced hippocampal reactive astrocytosis and significantly greater GFAP levels relative to WT mice. IGF-1 overexpression stimulated Akt phosphorylation and reduced acute (1 and 3d) hippocampal neurodegeneration, culminating in greater neuron survival at 10d after CCI injury. Hippocampal neuroprotection achieved by IGF-1 overexpression was accompanied by improved motor and cognitive function in brain-injured mice. These data provide strong support for the therapeutic efficacy of increased brain levels of IGF-1 in the setting of TBI.

## Introduction

Traumatic brain injury (TBI) is one of the major causes of death and disability worldwide. TBI survivors often experience cognitive impairment suggestive of neuronal damage in areas controlling learning and memory [1]. Hippocampal activity, critical for acquisition and retrieval of short-term memory tasks [2], [3], is compromised after TBI. Experimental models of TBI recapitulate learning and memory dysfunction as well as altered long term-potentiation and cell loss in the hippocampus [4]–[8]. For example, controlled cortical impact (CCI) brain injury results in spatial memory impairment and neuronal damage in the cornu ammonis-3 (CA-3), CA-1 and dentate gyrus subregions, the severity of which can be controlled by altering the depth of impact [9], [10].

Owing to their pluripotent actions in the central nervous system (CNS), neurotrophic factors are considered potential therapeutic agents for TBI [11]. Insulin-like growth factor-1 (IGF-1) is a 7.5-kDa polypeptide hormone that in the CNS acts as a neurotrophic factor, essential for neural cell differentiation, proliferation and survival during development and adulthood. IGF-1 mediates its anabolic effects through the IGF-1 receptor (IGF-1R) that is linked to two major signaling pathways, PI3K/Akt and MAP kinase [12]. Studies suggest the PI3K/Akt pathway predominates in IGF-1 mediated neuroprotection [13]–[15]. In the adult brain IGF-1 expression is low compared to the developing brain and is mainly present in neurons [16]. Endogenous levels of IGF-1 increase transiently after TBI [17], but may be insufficient to sustain damaged neurons. Although administration of exogenous IGF-1 confers neuroprotection in experimental models of ischemic brain injury [18]–[21], its effectiveness in promoting cell survival after TBI is largely unknown. Nonetheless, systemic administration of IGF-1 as a potential therapy for TBI is supported by studies demonstrating improved behavioral outcomes in rodent models [22], [23] and by clinical studies demonstrating improved nitrogen balance in patients with severe TBI [24].

To determine the neuroprotective role of IGF-1 in TBI in the absence of potential systemic effects, we used previously characterized IGF-1 transgenic (IGF-1Tg) mice [25]. IGF-1 overexpression was restricted to glial fibrillary acidic protein (GFAP) expressing cells with transgene expression under the control of a ‘Tet-off’ system. The ‘Tet-off’ strategy allowed suppression of IGF-1 overexpression during postnatal development by administration of doxycycline. Because contusion TBI produces astrogliosis accompanied by increased GFAP expression in regions of neuronal damage, we postulated that GFAP-linked IGF-1 overexpression would effectively deliver IGF-1 to vulnerable brain areas.

Utilizing a targeted IGF-1 delivery technique linking IGF-1 overexpression to regional posttraumatic reactive astrocytosis, we show that following moderate or severe contusion brain injury, astrocyte-derived IGF-1 exerts autocrine effects on astrocytes, reduces regional hippocampal neurodegeneration and improves post-traumatic cognitive and motor function.

## Materials and Methods

### Animals

To generate astrocyte-specific IGF-1 transgenic mice with conditional overexpression, heterozygous tTA^GFAP^ mice were bred with heterozygous IGF-1^pTRE^ mice [25]. Detailed characterization of IGF-1 overexpressing mice was published previously [25]. Briefly, tTA^GFAP^ mice carry the tTA^GFAP^ transgene, obtained by linking the GFAP promoter to tetracycline-controlled transactivator protein (tTA) cDNA (Figure S1). The IGF-1^pTRE^ transgene was generated using cDNA coding for rat somatostatin signal peptide/human IGF-1 fusion protein that is inserted into a pTRE plasmid. By crossing tTA^GFAP^ mice with IGF-1^pTRE^, double transgenic mice carrying both the transgenes (tTA^GFAP^/IGF-1^pTRE^ ) were generated. In these double transgenic mice tTA is expressed selectively in astrocytes where binding to TRE drives IGF-1 expression. When doxycycline is provided, it binds to tTA preventing tTA-TRE binding, and hence hIGF-1 expression (Figure S1). From here on, the term IGF-1 transgenic (IGF-1Tg) is used to describe the double transgenic mice in which astrocyte-specific overexpression of IGF-1 can be suppressed by doxycycline. Wild-type (WT) littermates were utilized as controls in all studies.

Mice were fed with doxycycline supplemented mouse chow (200 mg/kg) for the first 4 weeks of postnatal development to block hIGF-1 transcription. Subsequently they received standard mouse chow for at least four weeks to allow transgene expression prior to surgery/injury. hIGF-1 mRNA was previously shown to be induced in the hippocampus after 3–4 weeks of doxycycline withdrawal [25]. A subset of mice in the doxycycline control study remained on the doxycycline diet from birth until euthanasia. Mice were housed at a maximum of 5/cage in a University of Kentucky Medical Center animal vivarium at a constant temperature (23±2°C) with a 14/10-h light/dark cycle and provided food and water *ad libitum.* All procedures involving animals were approved by the University of Kentucky’s Institutional Animal Care and Use Committee (approval # 2007–0191).

### Controlled Cortical Impact Injury

The CCI injury was performed as previously described [9], [17]. Anaesthesia was induced using 3% isoflurane. After securing the head of the animal in a stereotaxic frame (David Kopf Instruments, CA), anaesthesia was maintained using 2.5% isoflurane delivered through a nose cone. A midline scalp incision was made and a 5-mm diameter craniotomy was performed over the left parietal cortex, lateral to the sagittal suture (1.5 mm lateral) midway between Bregma and lambda (approximately −1.9 mm Bregma). Sham-injured mice received a craniotomy under isoflurane anesthesia. For CCI injury, a cortical contusion was produced using a pneumatically driven impactor device (Precision System Instruments, KY) with a 3 mm diameter rounded impactor tip. The impact depth was set at either 0.5 mm or 1.0 mm, with a velocity of 3.5 m/s to produce moderate or severe brain injury, respectively, as previously demonstrated using histological and behavioral endpoints [9], [26], [27]. Histological changes observed after 0.5 mm or 1.0 mm CCI were consistent with our previous observations [9]. Animals were randomly assigned to receive either sham injury (*n* = 4–6/genotype for histology, immunohistochemistry, western blotting and ELISA; *n* = 8/genotype for behavioral studies), or CCI brain injury (*n = *8/genotype for each time point for histology, immunohistochemistry; *n* = 5–8/genotype for each time point for western blotting and ELISA; *n* = 13/genotype for behavioral studies). After CCI or sham injury, a circular disk made from dental cement was glued over the craniotomy to protect the brain surface, and the scalp was sutured. Mice were placed on a Hova-Bator Incubator (model 1583, Randall Burkey Co., TX) to maintain body temperature until they regained consciousness, after which they were returned to their home cages.

### Tissue Preparation for Immunohistochemistry and Histology

Animals were deeply anesthetised by sodium pentobarbital at 72 h after 0.5 mm CCI or 24 h, 72 h or 10d after 1.0 mm CCI and transcardially perfused with heparinised saline followed by 10% buffered formalin. Brains were removed 24 h after post-fixation in 10% formalin, cryoprotected using 30% sucrose solution, and snap frozen in cold isopentane (≤−25°C). Frozen brains were cut in a coronal plane at 40 µm thickness.

### Immunohistochemistry

Immunohistochemistry was performed using published protocols for frozen sections [17]. For IGF-1 labeling, antigen retrieval was performed using 10 mM citric acid pH 6.0 at 60°C. Primary antibodies used were anti-IGF-1 (rabbit polyclonal,1∶1000, National hormone and peptide program, CA) anti-GFAP (mouse monoclonal,1∶1000, Sigma, MA) and anti-NeuN (1∶1000, Millipore, MA). Secondary antibodies were conjugated with biotin (Jackson Immunoresearch, PA) and, for amplification of the signal, avidin-biotin-enzyme complex (Vector Laboratories, CA) was used. For co-localization, sections were incubated with anti-IGF-1 (1∶500), anti SOX-2 (rabbit polyclonal, 1∶500, Millipore) or anti-p-Akt (rabbit monoclonal, 1∶300, Cell Signalling Technology, MA) in combination with either anti-NeuN (1∶500, Millipore) to label neurons or anti-GFAP (1∶300) to label astrocytes. Secondary antibodies were conjugated with Alexa-488, Cy-3 or Alexa-594 (Invitrogen, CA). Omission of primary antibody served as a negative control. Slides were observed and imaged using an epifluorescence microscope (AX 80 Olympus, PA), a spinning confocal microscope (IX-81, Olympus) equipped with a CCD camera or a Nikon Eclipse Ti-C2 confocal microscope. Confocal images were acquired as Z-stacks (0.5 µm thickness) and the representative image is a maximum intensity projection image from the Z-stack. Images obtained from the spinning confocal microscope were modified using the Image Pro flatten filter to reduce the intensity variations in the background pixels.

### Tissue Preparation for ELISA and Western Blotting

At 24 or 72 h after brain injury, mice were euthanized by CO_2_ asphyxiation and brains were rapidly removed onto an ice cold dissection plate. Contralateral and ipsilateral hippocampi were dissected and homogenized separately in chilled lysis buffer (1%Triton, 20 mM Tris–HCl, 150 mM NaCl, 5 mM EGTA, 10 mM EDTA, 10% glycerol, protease inhibitors) and centrifuged at 10,000 g for 30 min at 4°C. The supernatants were collected for ELISA and western blotting. Protein concentrations were determined using a BCA protein assay kit (Pierce Biotechnology, IL).

### Quantification of hIGF-1 by ELISA

IGF-1 ELISA was performed using Quantikine® human IGF-1 ELISA kit (R&D Systems Inc., MN) according to manufacturer’s directions. Briefly, hippocampal samples were pretreated to dissociate IGF-1 from IGF binding proteins (BPs). Pretreated samples and hIGF-1 standards (0.094–6.0 ng/ml) were then pipetted in duplicate onto a microwell plate coated with a monoclonal antibody specific to hIGF-1. After 2 h incubation the unbound antigens were removed by rinsing. HRP enzyme-linked polyclonal antibody specific to hIGF-1 was then added to the wells and incubated for 1 h. The wells were rinsed and substrate solution was added for 30 min. Development was stopped by adding 1N hydrochloric acid. Optical density (OD) was recorded using a microplate reader (Tecan, NC) at 450 nm with a reference filter of 540 nm. Readings at 540 nm were subtracted from OD at 450 nm to correct for background.

### Western Blot

The western blot analyses were performed as previously described [17]. Electrophoresis was performed using 10–30 µg of protein extracts on a 3–8% Tris-acetate gel at 150V and transferred onto nitrocellulose membranes. The membranes were blocked for 1 h in 5% dry milk and then incubated overnight with primary antibody, followed by 1 h incubation in secondary antibody. The antibodies used were anti-p-Akt ser ^473^ (rabbit monoclonal, 1∶2000, Cell Signaling Technology), anti-Akt (rabbit monoclonal, 1∶2000, Cell Signaling Technology), anti-GFAP (1∶5000) and anti–β-actin (mouse monoclonal, 1∶5000, Calbiochem Inc, CA). Secondary antibodies were conjugated to an infrared dye (1∶8000 IRDye800CW, Rockland, PA). After washing, the membranes were imaged and quantified using a Li-Cor Odyssey Infrared Imaging System (Li-Cor, NE). Following p-Akt^ser^ and GFAP development, the blots were reprobed for actin. All samples were run in duplicate. For quantification, the OD of each band was divided by its respective actin OD and then normalized to the mean relative OD for WT sham mice to allow comparison across gels.

### Fluoro-jade C Staining and Quantification of Hippocampal Neuronal Death

For each brain, every tenth section (400 µm apart) spanning the region of the cerebrum that contains hippocampus (Bregma levels −1.0 mm to −3.5 mm) was mounted and air dried onto gelatin-coated slides. Fluoro-jade C staining for degenerating neurons [28] has been used to quantify trauma-induced hippocampal neuron death [29], [30]. Staining was performed according to manufacturer’s directions. Briefly, slides were treated with 1% NaOH for 5 min followed by 2 min each in 80% ethanol, 70% ethanol, and distilled water. Slides were then placed in a 0.06% potassium permanganate solution for 10 min and rinsed in distilled water, before staining for 10 min with 0.0001% Fluoro-jade C (Millipore) in 0.1% acetic acid. Slides were then rinsed in distilled water, dried on a slide warmer, immersed in Xylenes, and coverslipped in Cytoseal XYL (Richard-Allan Scientific, MI). All slides were stained in the same experimental batch. For quantification, the entire dentate gyrus (DG) and CA-3 areas of the hippocampus from each section were imaged at 20X magnification using an epi-fluorescence microscope equipped with a FITC filter (AX80, Olympus). Exposure time and sensitivity were kept constant in all the photos. Multiple images were overlaid to create a high resolution montage of the entire DG or CA-3 (Adobe illustrator-CS4, Adobe systems Inc, CA). Using Image Pro Plus (Media Cybernetics, Inc, MD) software, an area of interest was selected by tracing around the granule cell layer of DG or the CA-3 pyramidal neuronal layer including CA-3c. A minimum intensity threshold was set using the background intensity of the staining and was kept constant for sections from the same brain. Size exclusion criteria were set at 3 µm^2^ to exclude fluorescent neuronal debris and 800 µm^2^ to exclude large non-neuronal dye accumulations if any. The number of Fluoro-jade C labeled cells for each region was averaged across all sections for a given animal.

### Nissl Staining

Every tenth section spanning the entire cerebrum was mounted and air dried onto gelatin-coated slides. The slides were rehydrated through graded ethanol solutions, immersed in water, stained with 0.5% Cresyl Violet (Acros Organics, NJ), dehydrated through graded ethanol solutions, cleared with Xylenes (Fisher Scientific, NJ), and mounted using Permount (Fisher Scientific).

### Motor Behavior Assessment

A modified neurological severity score (NSS), adapted from Tsenter and colleagues [31] was used as described by us previously [26] with minor modifications in the scoring paradigm to assess motor deficits and recovery of motor function at 1, 2, 3 and 4 days post-injury. At 24 h before brain injury, mice were acclimated to each of four elevated, 60 cm long Plexiglas beams of different widths (3.0, 2.0, 1.0 and 0.5 cm) and a 0.5 cm diameter wooden rod. After injury, each mouse received a maximum score of four if it successfully crossed the beam with normal forelimb and hindlimb usage and position. Three points were given for successfully crossing the beam despite either a forelimb or hindlimb hanging from the beam. Two points were given for crossing the beam with both a forelimb and hindlimb hanging from the beam. One point was given for crossing the beam despite inverting underneath the beam one or more times. If a mouse became inverted on the beam, it was righted and allowed to continue across. For the rod, each mouse received two points for successfully crossing the rod and one point was given for crossing despite inverting more than three times. A mouse received a score of zero if it did not move along or fell off of the beams or rod. NNS is given as a total score (maximum 18) obtained per mouse.

### Cognitive Performance Evaluation

Memory function was evaluated using a novel object recognition (NOR) paradigm [27], [31]. At 24 h prior to injury, mice were individually acclimatized for one hour to a clear plastic cage (32×20×14 cm) with an open top prior to the introduction for 5 min of two identical Lego® Duplo® minifigures placed in opposite corners of the cage. On day 7 after CCI, each mouse was re-introduced to its respective testing cage and allowed to explore freely for one hour. The two original objects were re-introduced to the cage and the time spent exploring each object was recorded over a 5 min period. After a 4 h delay, the mice were returned to their test cages where one of the original objects was replaced with a novel object. Time spent exploring each object was recorded for 5 min. A recognition index was calculated as the time spent exploring the novel object as a percent of total object exploration time.

### Stereological Counting of CA-3 Neurons

To estimate the total number of NeuN positive cells in hippocampal CA-3 region, unbiased stereological cell counting using the optical fractionator technique [32], [33] was performed at 10d post-injury on a cohort of mice randomly selected from those that received behavioral evaluations. For each brain, every 5th section (i.e. 200 µm intervals) from bregma levels −1.0 mm to −2.2 mm encompassing the dorsal hippocampus was NeuN immunostained. Counting was restricted to the dorsal hippocampus because CCI results in CA-3 loss predominantly in the dorsal hippocampus, and because changes in organization and neuronal packing density in the ventral hippocampus would require a different set of sampling parameters. Neurons in CA-3 area were visualized using a 60X oil immersion objective on an Olympus BX-51 microscope equipped with a motorized stage and stereology software (Bioquant Life Science, V8.40.20; Bioquant Image Analysis, Nashville, TN). Regions of interest were traced and overlaid with a counting grid (150×150 µm^2^ ) for the optical disector method. Neurons were counted within the set disector area (20×20 µm^2^). The total number of CA-3 neurons was estimated using the formula, Σ = total cells counted×1/ssf×1/asf×1/tsf, where ‘ssf’ is the section sampling fraction, ‘asf’ is area sampling fraction and ‘tsf’ is thickness sampling fraction.

### Dentate Hilus Neuronal Counts

Four sections per brain selected at 400 µm intervals spanning from Bregma −1.5 mm to −2.7 mm were used for counting NeuN positive neurons in the dentate hilus (DH) using Image Pro Plus software. For each section, a photo montage was created for the DH and size exclusion criteria applied as described in the Fluoro-jade C quantification section. Total numbers of neurons in four sections were calculated for each animal.

### Statistical Analyses

All quantification procedures and behavioral analyses were performed by an investigator blinded to the injury and genotype conditions. Data are presented as mean+standard error of mean (SEM). For hIGF-1 ELISA data, western blot quantification, and NeuN neuronal counts statistical significance among experimental groups was determined by one-way analysis of variance (ANOVA). Where appropriate, ANOVA was followed by Newman-Keuls *post-hoc* analyses. Fluoro-jade C cell counts were analyzed using t-tests. NOR and modified NSS data were analyzed using a Kruskal-Wallis ANOVA at a given time point, followed by Mann-Whitney U tests. Statistical tests were performed using Statistica (StatSoft Inc, OK) software. For all comparisons p<0.05 was considered statistically significant.

## Results

### Astrocyte-specific Overexpression of IGF-1

**Figure 1:**
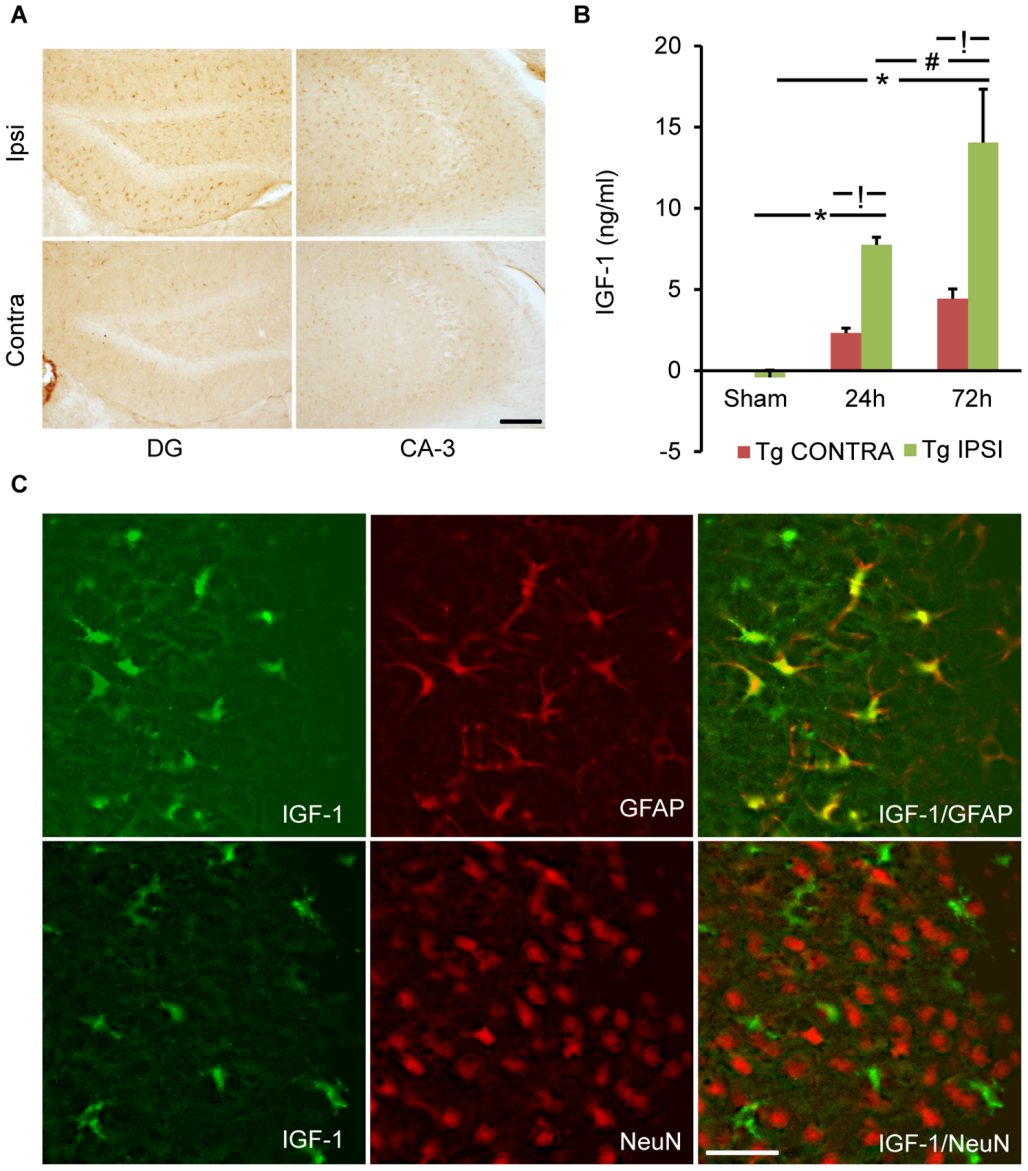
Trauma-induced astrocyte-specific IGF-1 overexpression. **A)** Coronal sections from IGF-1Tg mice show a marked increase in IGF-1 immunolabeling in the hippocampal dentate gyrus (DG) and CA-3 regions in the ipsilateral (ipsi) hemisphere compared to the contralateral (contra) after lateral controlled cortical impact (CCI) brain injury. Immunohistochemical staining was performed using an antibody that detects both human and mouse IGF-1. Representative picture is taken from an IGF-1Tg mouse 72 h after CCI. Scale bar = 100 µm. **B)** Quantification of IGF-1 expression using a human-specific IGF-1 ELISA. IGF-1 concentrations increased progressively in the ipsilateral hippocampus of IGF-1Tg mice at 24 h and 72 h after severe CCI brain injury. Data plotted as mean+SEM. * p<0.05 comparing injured with sham, **!** p<0.05 comparing ipsilateral to contralateral, and # p<0.05 comparing 72 h to 24 h. **C)** Confocal images taken from ipsilateral hemisphere bordering the contused cortex demonstrate widespread colocalization of IGF-1 (green) with the astrocyte-specific marker GFAP (red) in brain-injured IGF-1Tg mice. No co-localization of IGF-1 (green) and neuron-specific NeuN (red) was observed. Scale bar = 50 µm for C.

To verify astrocytic expression of IGF-1 we performed IGF-1 immunostaining in sham and brain-injured IGF-1Tg and WT mice, using an IGF-1 antibody that detects both mouse and human-IGF-1. Consistent with developmental downregulation of IGF-1 in the CNS, basal IGF-1 expression in the hippocampus of WT and IGF-1Tg sham-injured mice was low, with only weak immunoreactivity in cells with neuronal morphology (data not shown). Brain injury did not produce a noticeable change in IGF-1 expression in WT mice (data not shown). In contrast, after CCI brain injury, IGF-1 immunostaining was markedly increased in IGF-1Tg mice throughout the hippocampus ipsilateral to the impact in cells with an astrocytic morphology. Injury-induced increases in IGF-1 immunoreactivity in IGF-1Tg mice were even more prominent at 72 h (Figure 1A). Among the hippocampal subregions, the DG and CA-3 are known to be most susceptible to CCI brain injury [9]. In the DG, both the molecular layers and hilar region exhibited robust IGF-1 immunoreactivity (Figure 1A), while IGF-1 positive cells were absent from the DG granule cell layers. In comparison, only a few scattered IGF-1 positive cells with astrocytic morphology were observed in the contralateral hippocampus of brain- injured IGF-1Tg mice (Figure 1A). Post-traumatic induction of hIGF-1 expression was quantified using an ELISA specific for hIGF-1. While levels of hIGF-1 were undetectable in sham controls and in brain-injured WT mice (data not shown), CCI in IGF-1Tg mice produced a progressive increase in hIGF-1 levels from 24 to 72 h in the hippocampus ipsilateral to the impact (Figure 1B). The contralateral hippocampus exhibited smaller increases in hIGF-1 at 24 h and 72 h, reaching levels only approximately one third of those in the ipsilateral hippocampus (Figure 1B). In order to verify the cellular origin of IGF-1 expression in IGF-1Tg mice, dual immunofluorescence for IGF-1 and GFAP or NeuN was performed. Most GFAP-positive astrocytes (approximately 85%) were co-labeled with IGF-1, whereas NeuN-positive neurons did not co-express IGF-1, confirming astrocytic localization (Figure 1C).

### Astrocyte-selective IGF-1 Overexpression Promotes GFAP Upregulation and Astrocytic Hypertrophy

**Figure 2:**
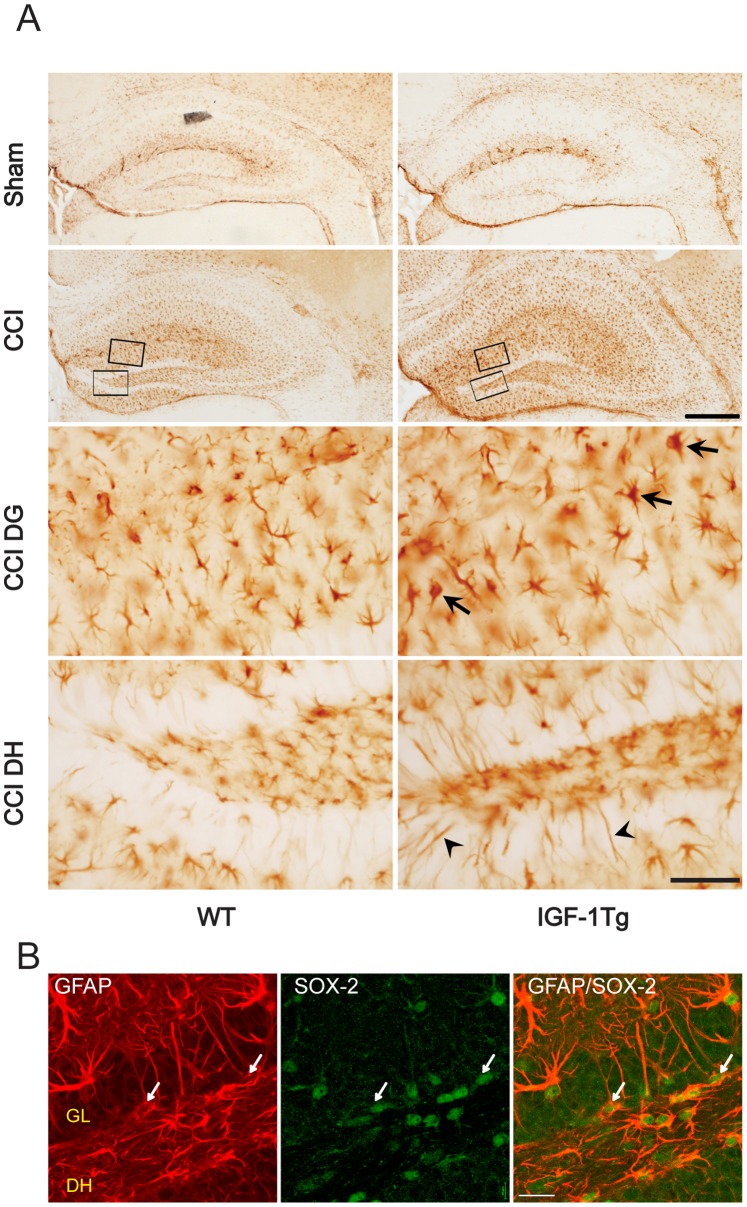
Enhanced astrocytosis in IGF-1Tg mice after brain injury. **A)** Immunoreactivity for GFAP was similar in the hippocampus of WT and IGF-1Tg sham mice. Controlled cortical impact (CCI) brain injury induced astrocytosis in the ipsilateral hippocampus which was more profound in IGF-1Tg mice than in WT mice (shown for 72 h, moderate severity). Boxed areas in the molecular layer of the DG and the apex of the dentate hilus (DH) are magnified in the two lower panels to illustrate hypertrophied astrocytes (arrows) and elongated astroglial processes (arrowheads). **B)** Representative confocal images from the DH of an IGF-1Tg mouse showing GFAP-stained processes arising from SOX-2 positive cells (arrows). DH: dentate hilus, GL: granule layer. Scale bars = 200 µm for low magnification images and 50 µm (A) or 100 µm (B) for high magnification images.

Basal astrogliosis, visualized using immunoreactivity for GFAP, appeared equivalent in WT sham and IGF-1Tg sham mice (Figure 2A). Brain injury resulted in robust reactive astrocytosis, indicated by increased GFAP staining, throughout the ipsilateral hippocampus at 24 and 72 h post-injury. However, trauma-induced astrogliosis was more pronounced in IGF-1Tg mice, characterized by greater astrocyte hypertrophy (Figure 2A). In addition, GFAP-positive cells within the subgranular layer or hilus of IGF-1Tg mice extended long processes through the DG granule layer, while WT mice typically exhibited little GFAP labeling within the granule layer (Figure 2A). To explore the possibility that these processes arose from radial glial stem cells, we performed dual immunofluorescence staining with antibodies against GFAP and SOX-2, a transcription factor commonly used as a stem cell marker. Many of GFAP-positive processes were found to be associated with SOX-2 positive nuclei (Figure 2B), raising the possibility that IGF-1 overexpression may alter stem cell as well as astrocyte GFAP expression. To confirm that this increased GFAP expression was specific to the genetic overexpression of IGF-1, we blocked IGF-1 overexpression by administering doxycycline (200 mg/kg feed) to IGF-1Tg mice from birth until euthanasia 72 h after induction of moderate brain injury. Doxycycline administration effectively prevented IGF-1 overexpression in the hippocampus as shown by a lack of IGF-1 immunostaining (Figure S2). In the absence of IGF-1 overexpression, injury-induced astrocytosis throughout hippocampus in transgenic mice was comparable to that observed in WT mice (Figure S2). These data strongly support that astrocyte-derived IGF-1 enhances injury-induced hippocampal astrocytosis and hypertrophy.

### IGF-1 Overexpression Increases GFAP Protein Levels

**Figure 3:**
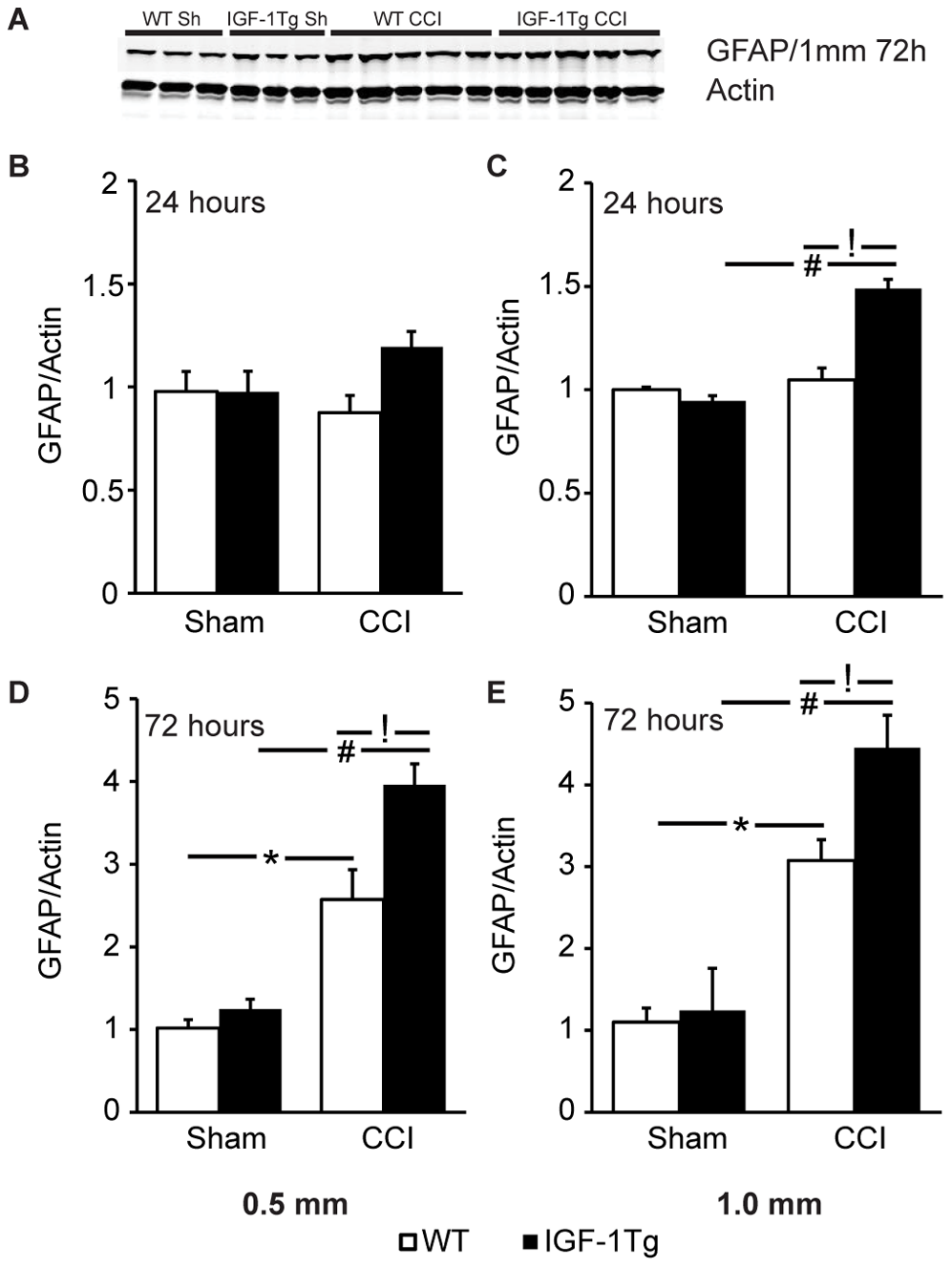
IGF-1 overexpression increased hippocampal GFAP levels following brain trauma. **A)** Representative western blot images for ipsilateral hippocampal samples probed with anti-GFAP and anti-actin antibodies. Blots illustrate expression at 72 h after severe controlled cortical impact (CCI) brain injury or sham injury (Sh). Relative expression of hippocampal GFAP at 24 h (**B,C**) and 72 h (**D,E**) following either 0.5 mm (**B,D**) or 1.0 mm (**C,E**) depth CCI in wildtype (WT, open bars) and IGF-1 transgenic (IGF-1Tg, closed bars) mice. Optical density from each band was normalised to its respective actin band and then group means were normalised to the mean of the WT sham group. Data represented as mean+SEM. * p<0.05 and # p<0.05 comparing CCI with sham, and **!** p<0.05 comparing IGF-1Tg to WT.

In order to quantify the effect of conditional astrocyte-specific IGF-1 overexpression on GFAP protein levels, we performed western blotting of the hippocampus after both moderate and severe brain injury. Consistent with immunohistochemical staining for GFAP (Figure 2A), WT and IGF-1Tg sham control mice had comparable GFAP levels (Figure 3). Brain-injured WT mice showed delayed upregulation of GFAP with significantly increased levels at 72 h, but not 24 h. At 72 h, moderate injury to WT mice resulted in a 2.5 fold increase in GFAP levels while severe injury produced a 3-fold increase (Figure 3D, 3E). IGF-1Tg mice exhibited an earlier increase in GFAP, but this increase at 24 h was statistically significant only after severe injury (Figure 3C). By 72 h post-injury, GFAP levels in the brain-injured IGF-1Tg mice were elevated approximately 4-fold above IGF-1Tg sham mice, and were significantly elevated above levels in WT brain-injured mice at both severities (Figure 3D, 3E).

### IGF-1 Overexpression Enhances Akt Phosphorylation

**Figure 4:**
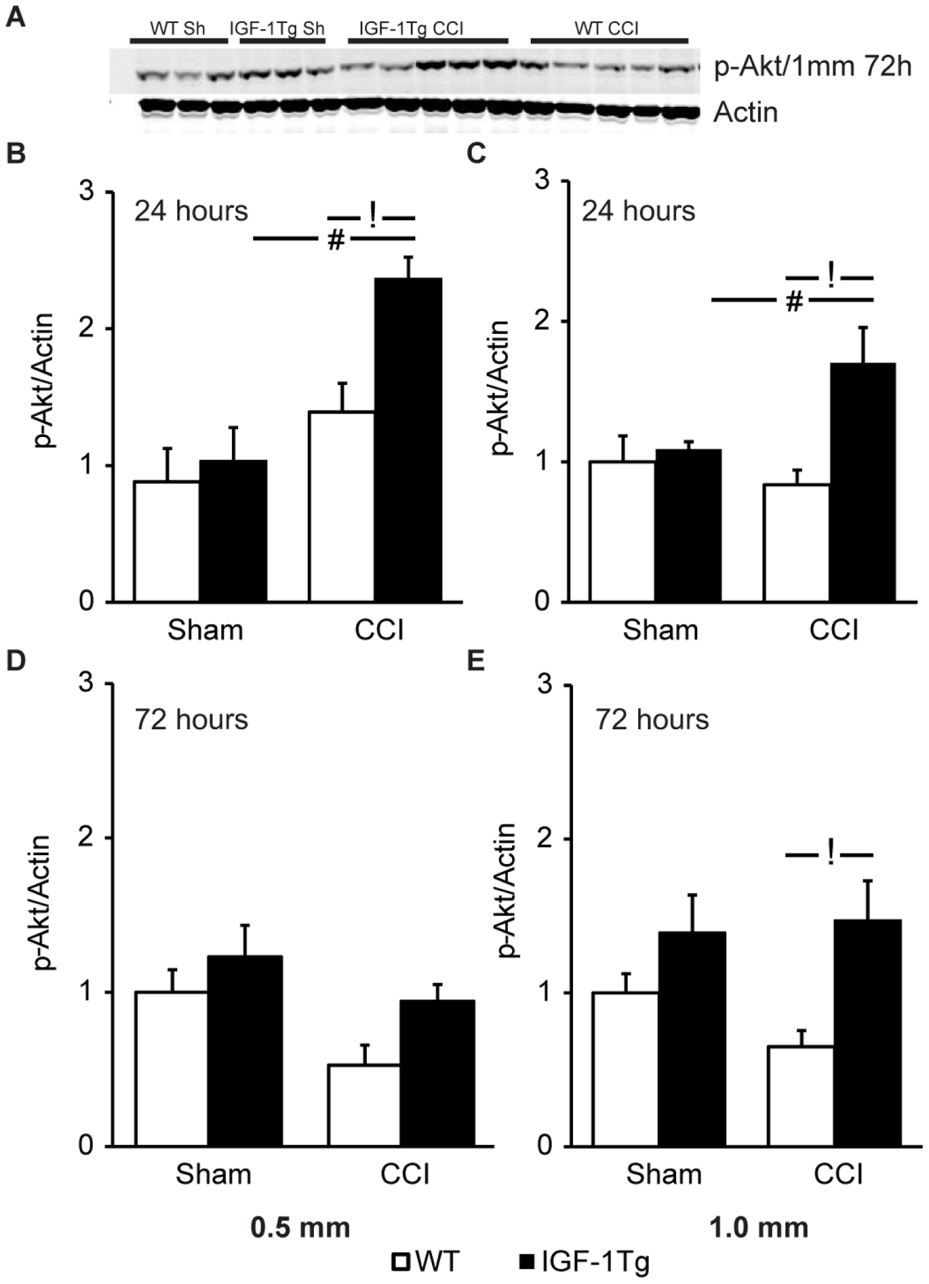
Akt phosphorylation is augmented in IGF-1Tg mice. **A)** Representative western blot images for ipsilateral hippocampal samples probed with anti-p-Akt and anti-actin antibodies. Blots illustrate expression at 72 h after severe (1.0 mm) controlled cortical impact (CCI) brain injury or sham injury (Sh). Relative expression of hippocampal p-Akt at 24 h (**B,C**) and 72 h (**D,E**) following either 0.5 mm (**B,D**) or 1.0 mm (**C,E**) CCI in wildtype (WT, open bars) and IGF-1 transgenic (IGF-1Tg, closed bars) mice. Optical density from each band was normalised to its respective actin band and then group means were normalised to the mean of WT sham group. Data are represented as mean+SEM. # p<0.05 comparing CCI with sham, and **!** p<0.05 comparing IGF-1Tg to WT.

In order to investigate the downstream signaling pathways of IGF-1, western blot analysis for p-Akt^ ser^ was performed. Hippocampal samples harvested at 24 or 72 h from WT and IGF-1Tg sham mice showed no statistically significant differences, although p-Akt^ ser^ levels appeared slightly elevated in IGF-1Tg sham mice at 72 h (Figure 4). Although brain injury appeared to result in a delayed decrease in p-Akt^ ser^ in WT mice by 72 h, levels were not significantly different from sham (Figure 4D, 4E). At 24 h following either moderate or severe CCI, IGF-1 overexpression significantly increased phosphorylation of Akt over levels in IGF-1Tg shams and brain-injured WT mice (Figure 4B, 4C). p-Akt levels were effectively maintained at baseline (sham) levels at 72 h in IGF-1Tg mice, countering the apparent decline in p-Akt^ ser^ in WT mice (Figure 4D, 4E). Indeed, in the severely injured IGF-1Tg group at 72 h, Akt phosphorylation was elevated compared to injured WT mice (Figure 4E). To identify the phenotype of cells expressing p-Akt, we labeled brain sections with p-Akt and either NeuN or GFAP. In the hippocampus of IGF-1Tg mice immunolabeling for p-Akt was localized to neurons (NeuN) and a subset of astrocytes (GFAP) (Figure S3).

Quantification of total Akt protein levels by western blotting revealed no differences among sham or brain-injured hippocampal samples from WT and IGF-1Tg, at 24 or 72 h after moderate or severe CCI (Figure S4), confirming that changes in pAkt protein levels were indicative of changes in Akt activation.

### Selective Overexpression of IGF-1 in Astrocytes Promotes Hippocampal Neuronal Survival

**Figure 5:**
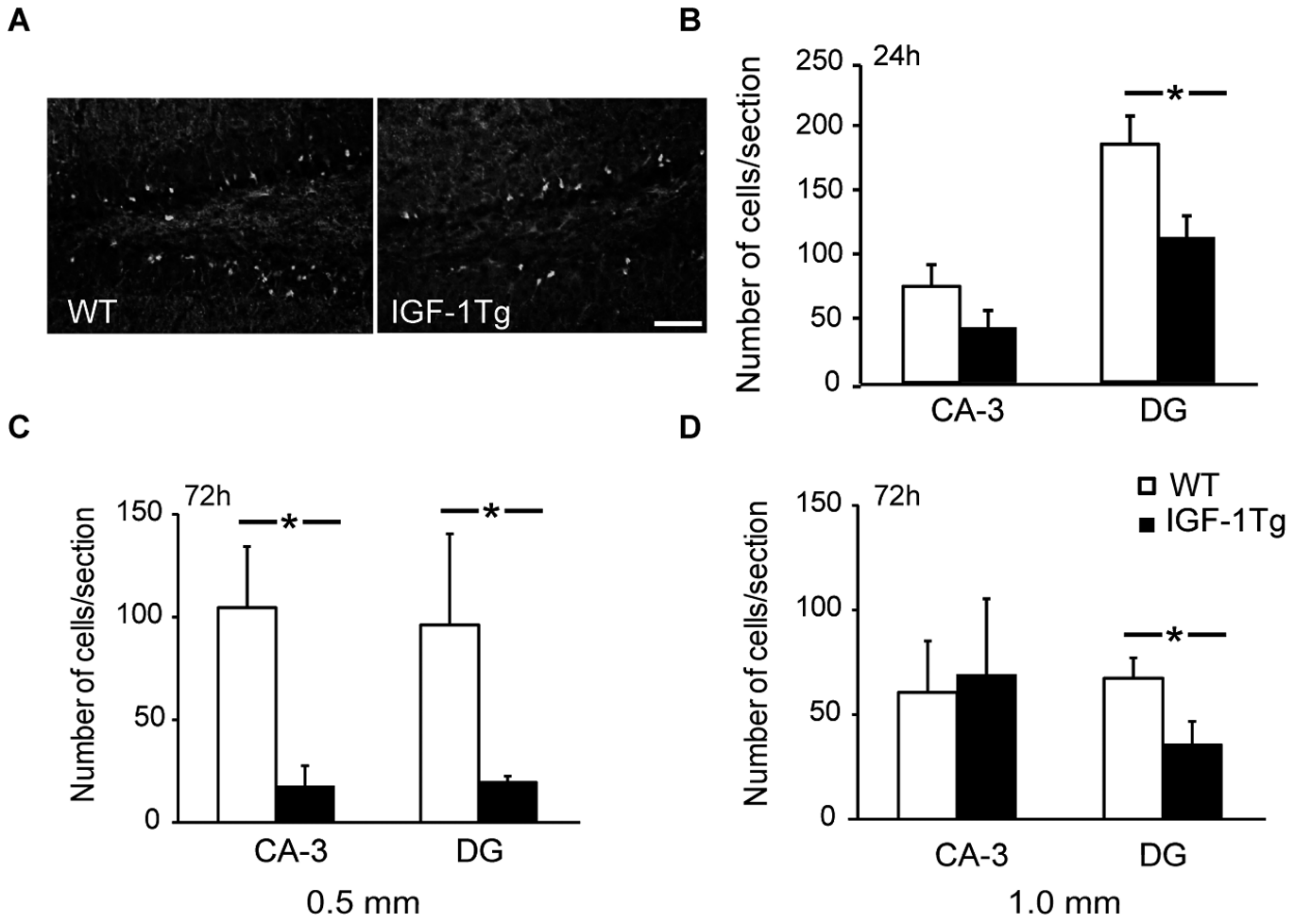
IGF-1 overexpression attenuated hippocampal neurodegeneration after trauma. **A)** Fewer Fluoro-jade C positive neurons were observed in the dentate gyrus (DG) of IGF-1 transgenic (IGF-1Tg) mice compared to wildtype (WT) mice at 72 h after cortical impact. Representative pictures are from severely (1.0 mm) injured WT and IGF-1Tg mice. Scale bar = 100 µm. Fluoro-jade C positive neuronal counts from ipsilateral hippocampal subregions CA-3 and DG after 0.5 mm **(C)** or 1.0 mm **(B, D)** cortical impact. Data are represented as mean+SEM. * p<0.05 comparing IGF-1Tg and WT.

CCI brain injury results in acute hippocampal neurodegeneration that can be detected using Fluoro-jade C. After either moderate or severe brain injury, Fluoro-jade C positive neurons were located predominantly in the hippocampal DG and CA-3 areas. Within the DG, many Fluoro-jade C positive neurons were located in the inner granular layer and subgranular zone (Figure 5A). Following severe CCI, numbers of Fluoro-jade C positive neurons in the ipsilateral hippocampal DG were significantly lower in IGF-1 overexpressing mice at both 24 h and 72 h (Figure 5B, 5D). However, in the CA-3 region, numbers of degenerating neurons were only modestly decreased in IGF-1Tg mice at 24 h (Figure 5D). At 72 h following moderate CCI, IGF-1 overexpression resulted in robust protection against neuronal death in both the CA-3 and DG (Figure 5C). No Fluoro-jade C stained neurons were observed in the hippocampus of sham-injured mice regardless of genotype.

**Figure 6:**
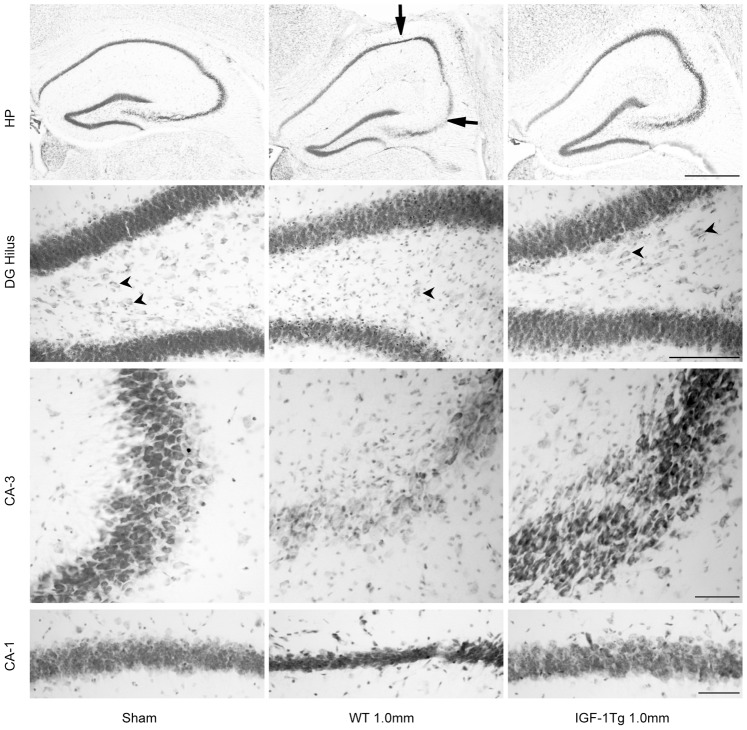
IGF-1 overexpression promoted hippocampal neuronal survival at 72 h after severe brain injury. Representative images of the ipsilateral hippocampus (HP) from Nissl-stained brain sections illustrate pallor or thinning of CA-3 and CA-1 areas of pyramidal layer (arrows) in wildtype (WT) mice. Higher magnification images from DG, CA-3 and CA-1 areas demonstrate hilar and CA-3 neuronal loss and thinning of the CA-1 pyramidal layer. IGF-1 overexpressing (IGF-1Tg) mice showed marked neuroprotection in each hippocampal subregion. Arrowheads point to hilar neurons. Scale bars = 500 µm (top HP panel), 100 µm for DG panel, 50 µm for CA-3 and CA-1 panels.

Because hippocampal neurodegeneration assessed using Fluoro-jade C staining captures neuronal degeneration ongoing at the time of euthanasia, Nissl staining was also used to evaluate cumulative neuron survival over the post-traumatic period. Severe (1.0 mm) brain injury in WT mice resulted in few Nissl-stained hilar cells with normal neuronal morphology, loss of Nissl staining in the CA-3 pyramidal layer, and thinning of the CA-1 pyramidal layer and DG granule layers at 72 h post-injury (Figure 6). Qualitative analyses indicated that IGF-1 overexpression provided protection against cell loss in CA-1, CA-3 and DG hippocampal areas (Figure 6). Following moderate (0.5 mm) CCI, surviving hilar neurons were more abundant in IGF-1 overexpressing mice than in WT mice (Figure S5), consistent with a neuroprotective effect IGF-1.

**Figure 7:**
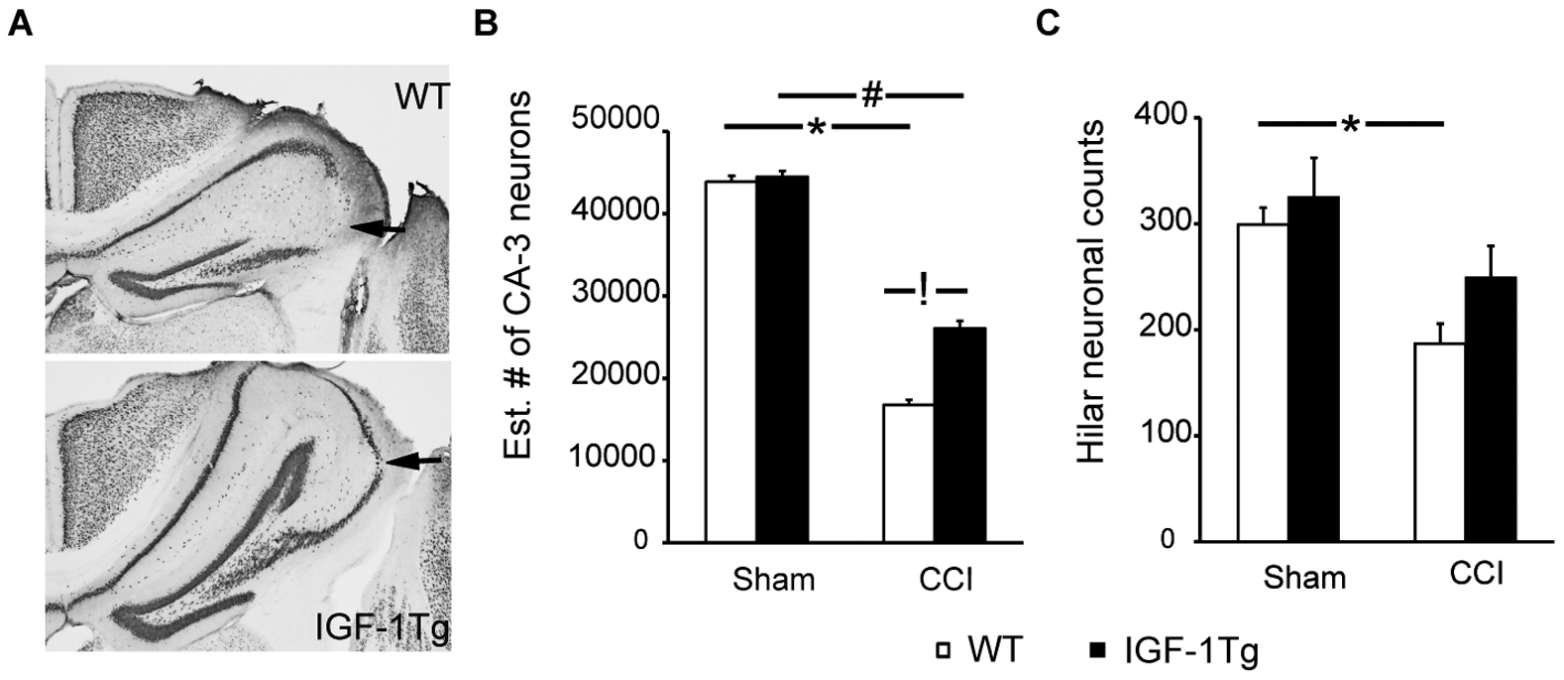
IGF-1 overexpression provided hippocampal neuroprotection 10d after severe brain injury. **A)** Representative images of the ipsilateral hippocampus from NeuN-immunostained brain sections after severe (1.0 mm) brain injury showing CA-3 neuronal loss (arrow). Scale bar = 200 µm. **B)** Stereological estimate of total number of CA-3 pyramidal neurons within the dorsal hippocampus (−1.0 to −2.2 mm Bregma) and **C)** Dentate hilar neuronal counts (total number of cells in 4 sections/animal) from wildtype (WT, open bars) and IGF-1 transgenic (IGF-1Tg, closed bars) mice. IGF-1 overexpression significantly increased neuronal survival in both regions. Data are expressed as mean+SEM. * p<0.05 and # p<0.05 comparing CCI with sham, and **!** p<0.05 comparing IGF-1Tg to WT.

To determine if IGF-1 hippocampal protection was sustained and to provide quantitative assessment of neuron survival, a separate cohort of IGF-1 Tg and WT mice received severe CCI and cell counting was performed at 10d post-injury in the CA-3 pyramidal layer and the dentate hilus (Figure 7). Numbers of NeuN-positive neurons were significantly decreased in brain-injured WT mice in the CA-3 (60% neuron loss, Figure 7B) and dentate hilus (30%, Figure 7C). IGF-1 overexpression increased the number of surviving neurons in both the CA-3 (Figure 7B) and dentate hilus (Figure 7C) subregions of hippocampus at 10d post-injury. In sham-injured mice, numbers of neurons in both the CA-3 and dentate hilus were equivalent in WT and IGF-1Tg mice.

### IGF-1 Overexpression Improves Post-traumatic Behavioral Dysfunction

**Figure 8:**
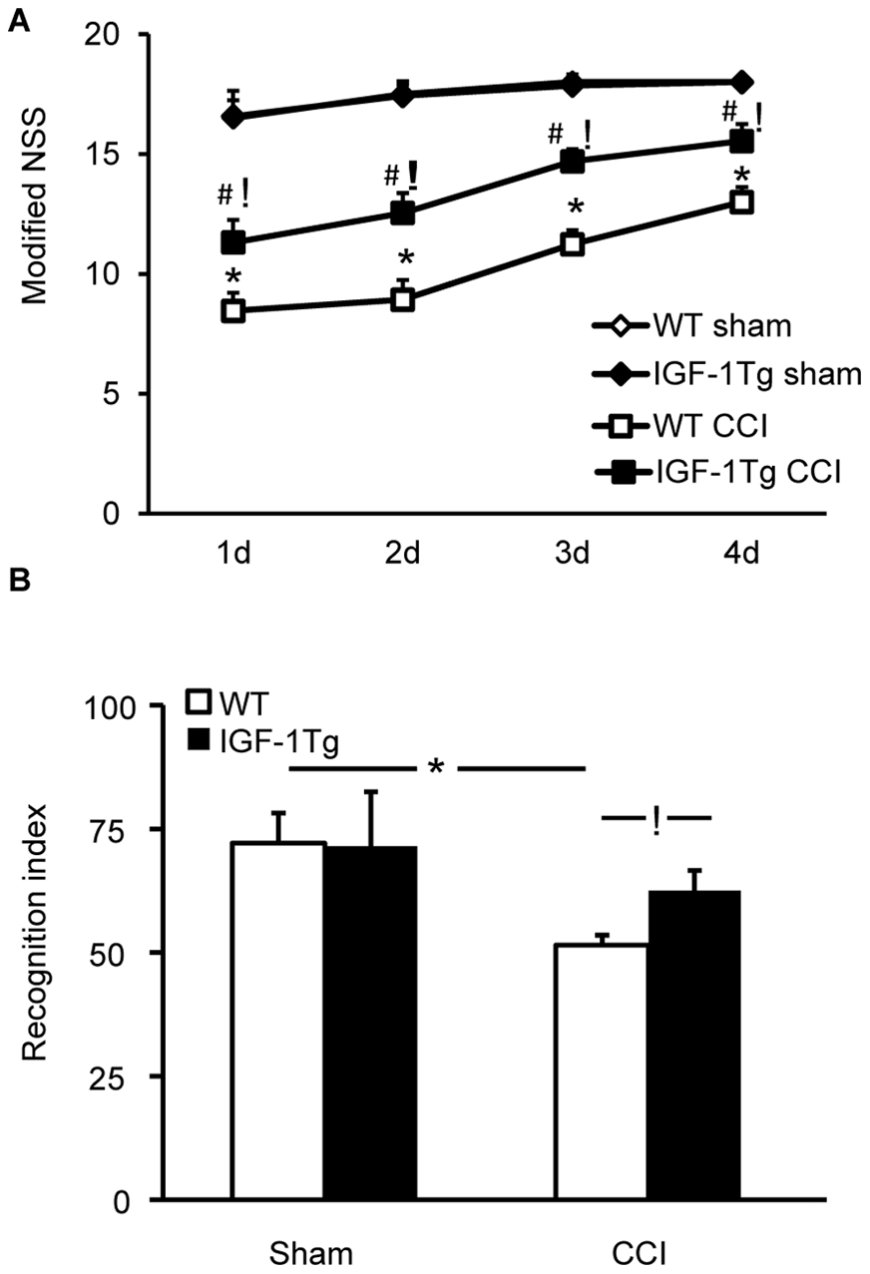
IGF-1 expression reduced post-traumatic behavioral impairment after severe brain injury. **A)** In the modified neurological severity score (NSS), a test series of beam walking tasks, IGF-1 overexpressing (IGF-1Tg) mice exhibited significantly better coordinated motor function compared to wildtype (WT) mice over the first 4d after severe (1.0 mm) CCI. **B)** In the novel object recognition (NOR) does task, the recognition index represent the percent exploration time spent on the novel object. IGF-1 overexpression prevented posttraumatic memory impairment at 7d post-injury. Data expressed as mean+SEM. * p<0.05 comparing WT CCI and WT sham, # p<0.05 comparing IGF-1 Tg CCI and IGF-1Tg sham, and ! p<0.05 comparing IGF-1Tg CCI to WT CCI.

To determine if the neuroprotection provided by IGF-1 overexpression was associated with behavioral improvement, motor function was evaluated by modified NSS and hippocampal-mediated cognitive performance by a NOR task at 7d post-injury. Brain injury resulted in significant coordinated motor function deficits as indicated by low NSS scores over the first four days after CCI in both WT and IGF-1Tg mice compared to their respective sham group (Figure 8A). However, injured IGF-1Tg mice achieved significantly higher NSS scores compared to injured WT mice at every time point assessed (Figure 8A), indicating that IGF-1 overexpression attenuated posttraumatic motor dysfunction. Motor performance of WT and IGF-1Tg sham mice was equivalent. Injury induced a significant impairment in memory in WT mice as revealed by a recognition index of 50%, indicative of no preference for a novel object over a familiar object (Figure 8B). Brain-injured IGF-1Tg mice did not exhibit a cognitive deficit relative to IGF-1Tg sham mice. Furthermore, brain- injured IGF-1Tg mice showed greater exploration of the novel object compared to brain-injured WT mice (Figure 8B), suggesting that IGF-1 overexpression improved memory of the familiar object.

## Discussion

In animal models of TBI, systemic IGF-1 administration improves behavioral outcomes [22], [23]. However, the mechanisms underlying IGF-1 mediated recovery of neurological function are not known. One of the many effects of IGF-1 in the CNS is promoting neuronal survival through activation of the PI3K/Akt pathway. Hence, we sought to investigate the neuroprotective potential of IGF-1 following brain injury focusing on the hippocampus, a structure vulnerable to trauma and critical for many aspects of learning and memory. Employing a targeted delivery technique that exploits reactive astrocytosis to deliver regionally specific IGF-1 in a delayed fashion after TBI, we demonstrate for the first time that IGF-1 provides hippocampal neuroprotection in addition to improving cognitive and motor function following brain injury. Our data also points to the activation of the pro-survival protein Akt as a potential mechanism underlying the neuroprotective effects of IGF-1.

Reactive astrocytosis typically occurs in a delayed fashion within and around areas of neuronal damage, with enhanced GFAP expression and glial scar formation progressing over several days [9], [34], [35]. Our western blot data demonstrates that in WT mice increases in hippocampal GFAP levels are delayed more than 24 h after CCI, although immunohistochemistry revealed reactive astrocytes by 24 h, consistent with previous studies [9], [35]. Linking hIGF-1 overexpression to GFAP expression was effective in producing a progressive increase in IGF-1 over 72 h that was three-fold higher in the ipsilateral compared to contralateral hippocampus, demonstrating that IGF-1 was targeted to areas of neuronal damage and death. Similar approaches using genetically modified astrocytes overexpressing BDNF or Nrf2 have proven beneficial in models of neurodegenerative diseases where astrocytosis is a hallmark of disease progression [36]–[38]. Measurements of IGF-1 at additional time points will be required to more fully elucidate the onset and duration of IGF-1 overexpression in brain-injured IGF-1Tg mice. However, based on the return of hippocampal GFAP mRNA levels to near baseline by 2 weeks after CCI brain injury [35], IGF-1 levels may also return to baseline by that time.

We previously showed that brain injury triggers a transient increase in cortical IGF-1 at 1 h followed by activation of p-Akt at 6 h that was not sustained [17]. In the present study, p-Akt^ser^ levels were unchanged at 24 h and decreased slightly at 72 h in WT mice. Together our data are consistent with a previous study of severely brain-injured rats where p-Akt^ser^ levels were increased at 2–6 h and reduced at 24–72 h in the hippocampus [39]. Overexpression of IGF-1 enhanced p-Akt^ser^ levels selectively in brain-injured mice. Sham-injured IGF-1Tg mice, in contrast, had p-Akt levels comparable to WT mice, confirming that Dox-mediated suppression of IGF-1 overexpression during first four weeks of postnatal development was sufficient to prevent basal increases in p-Akt levels reported with constitutive IGF-1 overexpression [40]. Posttraumatic IGF-1-mediated increases in p-Akt^ser^ were not due to an increase in Akt synthesis, as total Akt protein levels did not change. Although IGF-1 and GFAP levels increased progressively after CCI brain injury in IGF-1Tg mice, increases in p-Akt in brain-injured IGF-1 Tg mice were either comparable or smaller at 72 h compared to 24 h. This could be due, in part, to IGF-1 overexpression having to compensate for injury-induced decreases in p-Akt levels by 72 h. Furthermore, it is likely that turnover of activated Akt occurs much faster than that of GFAP, a structural protein; protein levels at 72 h may therefore represent cumulative increases to a greater extent for GFAP than for pAkt. Diminishing levels of Akt activation over time may also reflect decreased bioavailability of IGF-1 due to upregulation of IGF-binding proteins in response to prolonged IGF-1 overexpression.

In brain-injured mice, Akt was activated in neurons and astrocytes. Neurons are more susceptible to cell death when p-Akt levels are low, while neurons with enhanced Akt phosphorylation appear to be protected after CNS insults [41], [42]. PI3K/Akt signaling is the primary survival pathway initiated by IGF-1 in neurons and oligodendrocytes, leading to the inhibition of pro-apoptotic factors such as Bad, caspase-3, caspase-9, Forkhead transcription factors and NF-kB [43], [44]. While Akt activation may have contributed to the neuroprotective effect observed in injured IGF-1Tg mice, additional studies are necessary to establish a causal role for p-Akt or other downstream signaling molecules such as MAP-kinase [45]. Akt activation is also a key event in synaptic plasticity and the persistence of long-term potentiation [46], [47]. This may have relevance when considering the improved cognitive performance of IGF-1Tg mice.

The role of IGF-1 in preventing apoptotic death and promoting cell survival has been well established in *in vitro* studies [14], [15], [48]–[50]. Although IGF-1 appears neuroprotective in models of stroke and ischemic injury [51]–[54], this is the first study to assess neuroprotective actions of IGF-1 in a clinically relevant model of brain trauma. As previously reported for the CCI model [26], [55], [56], Fluoro-jade C positive dying neurons were mainly present in hippocampal subregions CA-3 and DG granule layers. IGF-1 overexpression provided notable protection against acute neurodegeneration in both areas after moderate severity injury. After severe contusion injury, IGF-1 significantly reduced acute DG neuron death at 24 and 72 h post-injury while it appeared to lessen CA-3 neurodegeneration only at 24 h. However, counts of surviving neurons at 10d post-injury confirmed that IGF-1 overexpression was neuroprotective in both the CA-3 and DG regions after severe contusion injury. The discrepancy between IGF-1 effects on acute CA-3 neurodegeneration and longer term CA-3 neuron survival may reflect limitations in quantifying cell death utilizing Fluoro-jade C, which provides a ‘snapshot’ of dying cells and may not convey sufficient information about alterations in the time course of neurodegeneration. It is also possible that IGF-1 exerts region-specific effects. Following ex vivo hippocampal stretch insult, DG neurons show higher caspase-3 activation compared to CA-3 neurons, in which calpain activity dominated [57]. Because of its potent anti-apoptotic effects [48], [58], [59], IGF-1 could be more effective in rescuing DG neurons. Enhanced DG neuron survival may have important implications in light of published observations that Fluoro-jade C positive DG neurons are predominantly doublecortin-expressing immature neurons [56] and newborn neurons are thought to mediate cognitive recovery after TBI [60].

Higher GFAP levels and enhanced astrocytic hypertrophy were observed in brain-injured IGF-1Tg mice compared to WT mice suggesting that astrocyte-derived IGF-1 exerted autocrine as well as paracrine effects. Increased GFAP levels could reflect GFAP upregulation or increased glial proliferation. IGF-1 is a potent mitogen for neural cells, particularly astrocytes [20], [61] and oligodendrocytes [62], [63]. Although we did not examine the full time course of posttraumatic astrocytosis, it seems likely that reactive astrocytosis would be prolonged in IGF-1Tg mice compared to WT given the increased GFAP levels within the first 72 h in IGF-1Tg mice. The autocrine actions of astrocyte-derived IGF-1 might then contribute to a feed-forward loop in which increased GFAP expression drives continued increases in IGF-1 levels, prolonging not only reactive astrocytosis but also IGF-1 expression. Reactive astrocytosis has long been considered detrimental due to its roles in inhibiting axonal regeneration and releasing inflammatory cytokines [64], [65]. However, astrocytosis may also be beneficial after injury by forming a physical and biochemical barrier to separate a contused area from healthy tissue, limiting the spread of inflammatory molecules and cells [66], [67]. Indeed, removal of reactive astrocytes after TBI has been shown to worsen tissue loss and behavioral performance [68]. Astrocytosis involves an early hypertrophic response and a subsequent hyperplastic response [69]. It is interesting to note that the acute hypertrophy stage is considered responsible for the beneficial effects of astrocytosis [70] and that IGF-1 overexpression produced a more pronounced hypertrophic response.

In addition, astrocytes with long processes that resemble radial glia were observed in the DG of brain-injured IGF-1Tg mice. GFAP-positive astrocytes with a radial glia-like morphology that reside in SGZ are considered as stem cells [71]. Because radial glial stem cells also express SOX-2, we performed dual labeling of GFAP and SOX-2. GFAP positive astrocytes were associated with SOX-2 positive nuclei raising the possibility that they are stem cells residing in the SGZ. Effects of IGF-1 on radial glia-like cells in the hippocampal SGZ could point to a role for IGF-1 in brain remodeling following trauma. Effects of IGF-1 on post-traumatic neurogenesis are currently under investigation in our laboratory. Thus additional studies are warranted to determine if enhanced neuronal survival and improved behavioral function after TBI in IGF-1 overexpressing mice is due to direct effects of IGF-1 on neurons or indirect effects mediated through astrocytes.

Cognitive and motor improvements have been observed in brain-injured rodents receiving systemically administered IGF-1 [22], [23]. Although exogenous IGF-1 administration improves behavioral outcome in brain-injured rodents, previous studies did not examine a link between functional improvements and cellular neuroprotection. Furthermore, systemic delivery of IGF-1 can be associated with undesirable side effects such as weight gain, hypoglycemia and tumorigenesis due to IGF-1’s effects on multiple organ systems [23], [24], [72]. Our targeted IGF-1 delivery technique demonstrates the direct CNS effects of IGF-1 on behavioral function. Improved novel object recognition in IGF-1Tg mice may be related to enhanced hippocampal neuronal survival, as hippocampal neuronal loss has been shown to correlate with cognitive deficits in brain-injured rodents [73], [74]. However, IGF-1 overexpression also attenuated motor dysfunction, consistent with previous observations of motor improvement following systemic IGF-1 administration in rats [23]. These findings raise the possibility that IGF-1 overexpression may be mediating effects in multiple brain regions. CCI brain injury is known to induce extensive astrocytosis not only in the hippocampus, but also in the cortex. If astrocyte-derived IGF-1 is neuroprotective in the cortex, this could contribute to improved motor function in IGF-1Tg mice after brain injury, as early impairment in motor function after TBI has been correlated with the volume of cortical injury [31]. The effects of IGF-1 overexpression on injury-related neuropathology in the cortex are currently under investigation.

In conclusion, enhancing IGF-1 expression by astrocytes provided hippocampal neuroprotection and improved memory and motor function after TBI. These data provide compelling evidence for the beneficial effects of CNS-derived IGF-1 for TBI treatment as well as the usefulness this cell-specific, gene-based approach in delivering IGF-1 specifically to vulnerable brain regions. Delivering IGF-1 through reactive astrocytes targeted IGF-1 overexpression to the damaged hippocampus, producing a progressive increase in IGF-1 over 72 h which led to activation of the Akt pro-survival pathway and reduced hippocampal neuron loss in multiple regions after both moderate and severe contusion TBI. Importantly, hippocampal neuroprotection was coupled with improved cognitive and motor function. This strategy may be suitable for delivery of IGF-1 and other endogenous molecules in other CNS disorders in which astrocytosis is a prominent pathological feature.

## Supporting Information

Figure S1 **Schematic representation of conditional overexpression strategy.** The M-1 mouse line carries a transgene for producing astrocyte-specific tetracycline transactivator protein (tTA), where the GFAP promoter (pGFAP) is linked to tTA. M-2 mice have a tetracycline responsive element (TRE) upstream of a CMV-hIGF-1 insert. By crossing M-1 and M-2 lines mice were generated that carry both transgenes (IGF-1Tg) in which tTA protein expressed only in GFAP-positive astrocytes binds to TRE to induce IGF-1 transgene expression. When doxycycline (DOX) is present it tightly binds to tTA, suppressing IGF-1 expression.
(TIF)

Figure S2 **Continuous Doxycycline (Dox) induced suppression of IGF-1 overexpression eliminates enhanced astrocyte hypertrophy in IGF-1Tg mice after brain injury.** WT and IGF-1Tg mice were maintained on Dox-supplemented chow to suppress IGF-1 overexpression during the entire period of study. At 72 h after moderate brain injury, IGF-1 immunostaining in IGF-1Tg mice was comparable to that in WT mice indicating that IGF-1 overexpression was blocked by Dox (top panels). Injury-induced reactive astrocytosis was also comparable in WT mice and IGF-1Tg mice (middle panels). Boxed areas are magnified in the bottom panels to illustrate similar GFAP immunoreactivity in hypertrophied astrocytes in the hippocampus ipsilateral to the impact. Scale bar = 100 µm (IGF-1 and GFAP panels) and 50 µm (high magnification lower panel).
(TIF)

Figure S3 **Akt phosphorylation in IGF-1Tg mice is associated with neurons as well as astrocytes.** Upper panels show representative confocal images from the ipsilateral hippocampus (CA-3 area shown) of brain-injured IGF-1Tg mice showing co-localization (arrows) of p-Akt (green) with the neuronal marker NeuN (red). Not all neurons expressed p-Akt (arrowheads). Lower panels show the dentate gyrus area where a subset of GFAP-positive (red) astrocytes clearly expressed p-Akt (green) (arrows), while many did not (arrowheads). DH: dentate hilus, GL: granule layer. Scale bar = 100 µm.
(TIF)

Figure S4 **IGF-1 overexpression did not change total Akt (T-Akt) protein levels after brain injury.** Representative western blot image showing total Akt (T-Akt) and actin from hippocampal homogenates of wild-type (WT) and IGF-1Tg mice at 72 h after 1.0 mm controlled cortical impact (CCI) or sham (Sh) injury.
(TIF)

Figure S5 **IGF-1 overexpression promoted hilar neuron survival at 72** **h after moderate brain injury.** Nissl staining (Cresyl violet dye) was performed to detect surviving neurons in the hippocampus (HP). At 72 h after moderate (0.5 mm depth) injury, the hilus of the dentate gyrus (DG) in wildtype (WT) mice exhibited partial neuronal loss. The density and morphology of Nissl-stained hilar neurons (arrowheads) was improved in brain-injured IGF-1 transgenic (IGF-1Tg) relative to WT mice. Scale bars = 500 µm (upper panel) and 100 µm (lower panel).
(TIF)
