# Supplementary figures and images for: Astrocyte-Specific Overexpression of Insulin-Like Growth Factor-1 Protects Hippocampal Neurons and Reduces Behavioral Deficits following Traumatic Brain Injury in Mice
Source: PLoS One. 2013 Jun 27;8(6):e67204. doi: 10.1371/journal.pone.0067204 (PMC3695083; doi:10.1371/journal.pone.0067204)

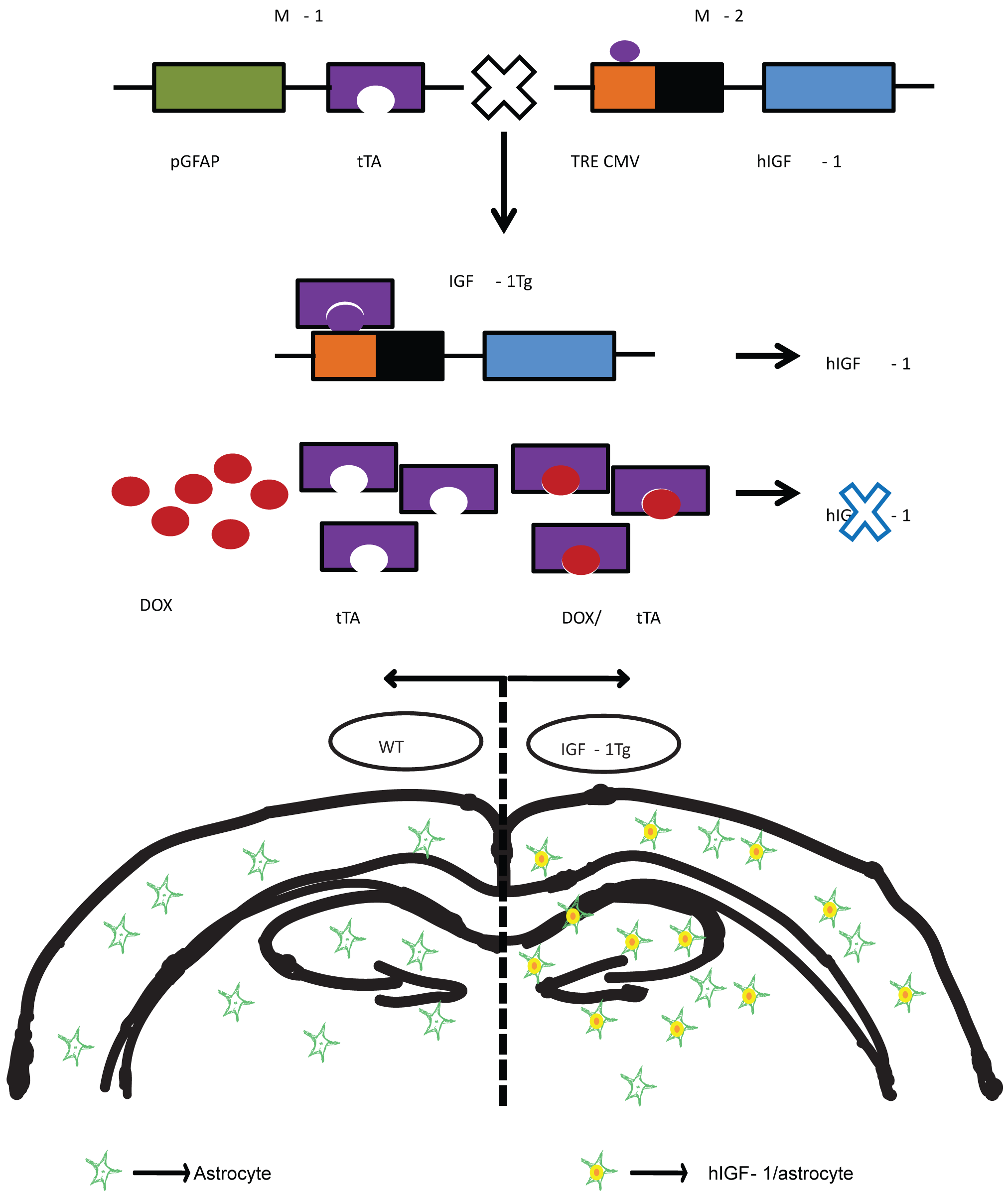

Supplement: Figure S1 — Schematic representation of conditional overexpression strategy. The M-1 mouse line carries a transgene for producing astrocyte-specific tetracycline transactivator protein (tTA), where the GFAP promoter (pGFAP) is linked to tTA. M-2 mice have a tetracycline responsive element (TRE) upstream of a CMV-hIGF-1 insert. By crossing M-1 and M-2 lines mice were generated that carry both transgenes (IGF-1Tg) in which tTA protein expressed only in GFAP-positive astrocytes binds to TRE to induce IGF-1 transgene expression. When doxycycline (DOX) is present it tightly binds to tTA, suppressing IGF-1 expression. (TIF) [file pone.0067204.s001.tif]

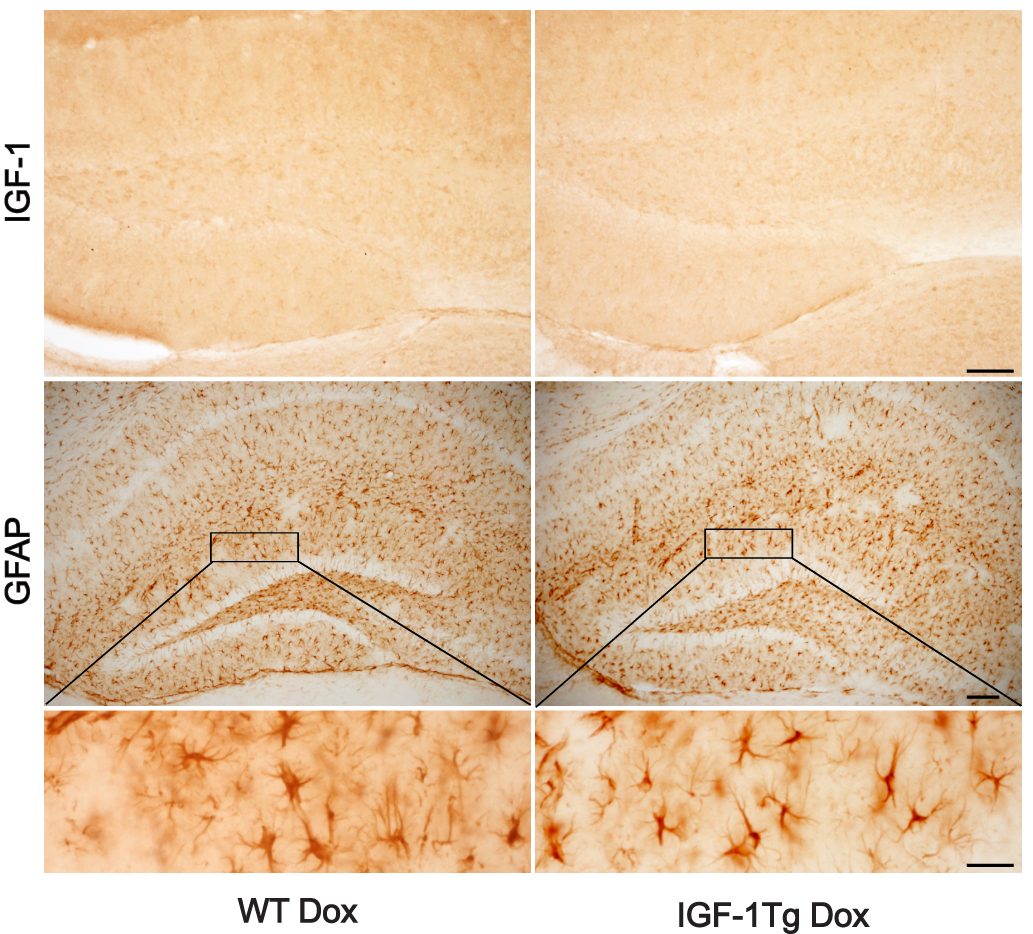

Supplement: Figure S2 — Continuous Doxycycline (Dox) induced suppression of IGF-1 overexpression eliminates enhanced astrocyte hypertrophy in IGF-1Tg mice after brain injury. WT and IGF-1Tg mice were maintained on Dox-supplemented chow to suppress IGF-1 overexpression during the entire period of study. At 72 h after moderate brain injury, IGF-1 immunostaining in IGF-1Tg mice was comparable to that in WT mice indicating that IGF-1 overexpression was blocked by Dox (top panels). Injury-induced reactive astrocytosis was also comparable in WT mice and IGF-1Tg mice (middle panels). Boxed areas are magnified in the bottom panels to illustrate similar GFAP immunoreactivity in hypertrophied astrocytes in the hippocampus ipsilateral to the impact. Scale bar = 100 µm (IGF-1 and GFAP panels) and 50 µm (high magnification lower panel). (TIF) [file pone.0067204.s002.tif]

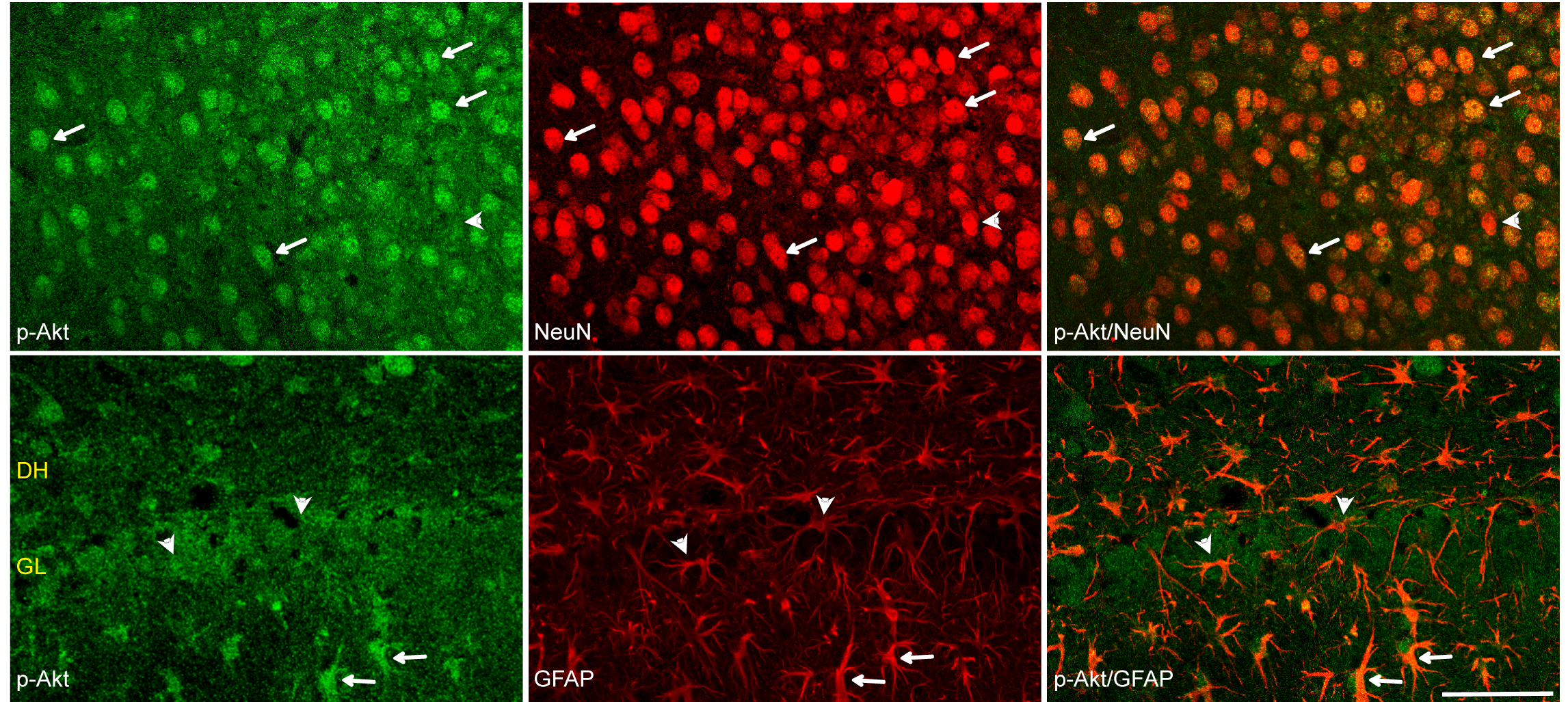

Supplement: Figure S3 — Akt phosphorylation in IGF-1Tg mice is associated with neurons as well as astrocytes. Upper panels show representative confocal images from the ipsilateral hippocampus (CA-3 area shown) of brain-injured IGF-1Tg mice showing co-localization (arrows) of p-Akt (green) with the neuronal marker NeuN (red). Not all neurons expressed p-Akt (arrowheads). Lower panels show the dentate gyrus area where a subset of GFAP-positive (red) astrocytes clearly expressed p-Akt (green) (arrows), while many did not (arrowheads). DH: dentate hilus, GL: granule layer. Scale bar = 100 µm. (TIF) [file pone.0067204.s003.tif]

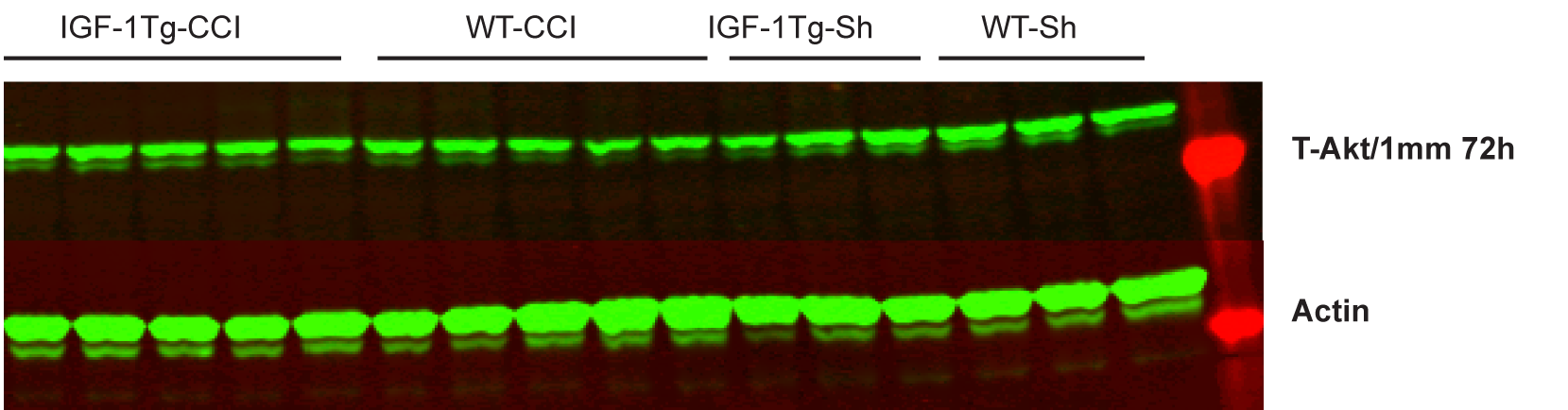

Supplement: Figure S4 — IGF-1 overexpression did not change total Akt (T-Akt) protein levels after brain injury. Representative western blot image showing total Akt (T-Akt) and actin from hippocampal homogenates of wild-type (WT) and IGF-1Tg mice at 72 h after 1.0 mm controlled cortical impact (CCI) or sham (Sh) injury. (TIF) [file pone.0067204.s004.tif]

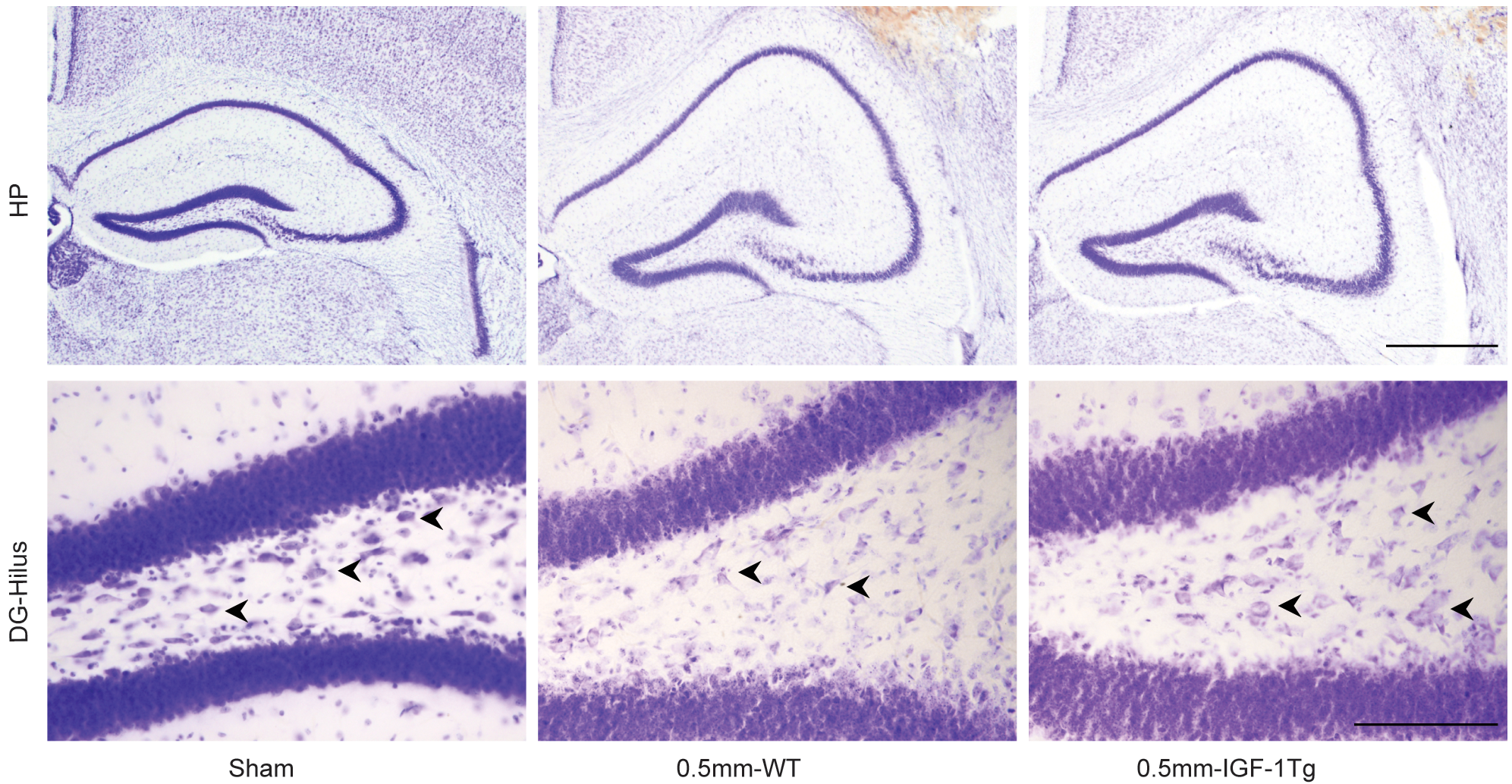

Supplement: Figure S5 — IGF-1 overexpression promoted hilar neuron survival at 72 h after moderate brain injury. Nissl staining (Cresyl violet dye) was performed to detect surviving neurons in the hippocampus (HP). At 72 h after moderate (0.5 mm depth) injury, the hilus of the dentate gyrus (DG) in wildtype (WT) mice exhibited partial neuronal loss. The density and morphology of Nissl-stained hilar neurons (arrowheads) was improved in brain-injured IGF-1 transgenic (IGF-1Tg) relative to WT mice. Scale bars = 500 µm (upper panel) and 100 µm (lower panel). (TIF) [file pone.0067204.s005.tif]
